# Slurry Tales: Newspaper Coverage of Livestock Slurry Reproduces Public Discourse on Agriculture in Germany

**DOI:** 10.1007/s00267-023-01798-w

**Published:** 2023-02-13

**Authors:** Astrid Artner-Nehls, Sandra Uthes

**Affiliations:** Leibniz Centre for Agricultural Landscape Research (ZALF), Müncheberg, Germany

**Keywords:** Manure, Media agenda setting, Media, Topic model, Text mining, Agriculture

## Abstract

The rapid transition of livestock husbandry in the 20th century involved a broad adoption of slurry-based livestock housing systems that resulted in farm economic benefits, but also in societal debate related to the environment and animal welfare. In this article, we apply the method of topic modeling to four major German newspapers to identify thematic emphases and changes in coverage around “slurry”. We considered more than 2300 articles published between 1971 and 2020. Our results show that reporting encompasses economic, environmental, and social topics in which slurry is represented mostly critically (“poisonous substance”), occasionally neutrally (“scent of countryside”), or rarely positively (“input for the bioeconomy”). Three meta-themes overarch the majority of issues and reflect public discourse on agriculture: (i) the dichotomy of agricultural industrialization and family farming; (ii) contrasting actualities of factory farming and animal welfare; and (iii) the responsibility of policy for the emergence, existence and solution of livestock and slurry-related problems. A more balanced recognition of mutual values and constraints by the media could contribute to a discursive reconciliation of public and private interests.

## Introduction

The vast majority of livestock in industrialized countries are kept in slurry-based housing systems, particularly dairy cows, fattening pigs, and breeding sows (Statistisches Bundesamt 2021). Slurry-based housing systems offer a number of management advantages that contributed to their fast adoption in the 20th century including their low labor and cost requirement, and scalability (Settele 2018). Since the 1970s, farm modernization schemes as well as agricultural extension and research promoted the new stabling systems (Albers 1999; Kleinschmidt 1984). The new husbandry systems enabled the keeping of previously unknown animal numbers with the lowest labor input ever. Yet the increased livestock supply also caused falling producer prices, which farmers tried to compensate through further intensification and expansion, a process well-known as technology treadmill (Cochrane 1958), which was further aggravated by price instruments such as meat export subsidies.

The expansion of livestock increasingly led to a mismatch between the amount of slurry and the fertilization needs of the farm fields, aggravated by the new availability of import feed in the 1970s causing a permanent overhang of nutrients imported to the farming systems, and legal changes in the 1980s, which decoupled the construction of stables from the availability of sufficient farm area (Deutscher Bundestag 2017). While the transformation was generally positively perceived as an enormous technological progress, it also caused new problems that society became only gradually aware of. Livestock slurry is often associated with increased emission and odor development, pollution, accidents and illegal disposal, and a livestock system, in which humans and animals are largely decoupled (Van der Ploeg 2003).

Selection and salience of issues by media, besides personal experience and information through social interaction, influence how livestock slurry is perceived by the audience (Neuman et al. 1992). What we perceive as “reality” is formed by contending discourses (Ryan 1991). Mass media both reproduce public discourses, and actively set the agenda. They are part of a pluralistic public sphere, in which manifold actors compete for attention. Journalists and editors function as gatekeepers who determine which information and informants will appear in the news (Shoemaker and Vos 2009) and “what an issue is about” (Pan and Kosicki 1993).

In this article, we aim to elucidate how “slurry” has been represented and framed in the German leading print media. We explore thematic emphases and changes in the degree of coverage over time. Such a long time horizon yields a large quantity of media articles (*n* > 2000), which is challenging to analyze. Therefore, we opted for a topic modeling approach, which is a suitable method for uncovering sematic patterns in large unstructured text corpora (Jiang et al. 2017).

A variety of studies that are concerned with media coverage of agricultural issues is available, particularly related to food issues, such as genetically modified organisms (e.g., Crawley 2007; Du and Rachul 2012; Fischer and Hess 2022; Lewison 2007; Morris et al. 2016) or health risks (Bateman et al. 2019; Feindt and Kleinschmit 2011; Gauthier 2011; Laestadius et al. 2012; Shan et al. 2014; Washer 2006), to ethics in animal farming (Buddle and Bray 2019), organic food (Danner et al. 2022; Meyers and Abrams 2010), bioenergy (Delshad and Raymond 2013; Sengers et al. 2010; Skjølsvold 2012; Wright and Reid 2011) as well as to representations of agriculture (Alföldi and Tutkun-Tikir 2008; Kayser et al. 2011; Wolfram et al. 2021).

Only few media studies on agricultural issues used text mining approaches (Artner-Nehls et al. 2022; De Brito et al. 2020) and even less topic modeling. Benites-Lazaro et al. (2018), for example, applied topic modeling to identify shifting public and media discourses of renewable energy and perceived impacts on food safety and climate change. Dehler-Holland et al. (2021) examined media coverage of the German Renewable Energy Act, and Danner et al. (2022) elucidated media agenda setting of organic food. However, there are no studies available that deal with the coverage of slurry in the media, and we intend to close this gap with this analysis.

Specifically, our research questions are:

RQ1: What are the thematic contexts (issues), in which slurry is reported on?

RQ2: How is slurry represented in these issues (positive, neutral, negative)?

RQ3: How do the issues develop over the investigation period?

RQ4: Does the coverage of slurry reflect public and political events?

## Material

For the topic model analysis of media coverage, we selected four German quality newspapers with nationwide dissemination: the two daily newspapers Frankfurter Allgemeine Zeitung (FAZ) and Süddeutsche Zeitung (SZ), the weekly newspaper Die Zeit (Zeit) as well as the weekly magazine Der Spiegel (Spiegel). These four news sources are characterized by comprehensive reporting (Zschache et al. 2010) and have a status as leading quality media, which makes them important agenda setters, that are widely used as sources for reporting by other journalists and intensively observed by political decision makers (cf. Robinson 2001). Daily newspapers publish more news than weekly magazines while weeklies offer more in-depth information and comments (Villnow et al. 2019). The two daily newspapers FAZ and SZ cover, besides national and international news, topics of the federated states Hesse respectively Bavaria they are situated in.

Table 1 gives an overview of the newspapers’ major characteristics and the number of articles we included in the analysis. We used the data from the digital archives of FAZ, Spiegel and Zeit from 1971 to 2020 and the digital archive of SZ that comprises articles only from 1994. In total, the text corpus consists of 2338 documents.

**Table 1:** Data source: newspapers, type, time span, number of articles, political orientation of newspapers

News sources	Type	Data availability	Number of articles	Political orientation
Süddeutsche Zeitung (SZ)	Daily newspaper (Mon–Sat)	1994–2020	754/754*	Center
Frankfurter Allgemeine Zeitung (FAZ)	Daily newspaper (Mon–Sun)	1949–2020	1127/990	Conservative
Der Spiegel (print edition)	News magazine (weekly)	1947–2020	252/239	Center-left
Die Zeit	Newspaper (weekly)	1946–2020	379/355	Center

*First number since the beginning of coverage, second number from 1971

### Topic Modeling

**Fig. 1:**
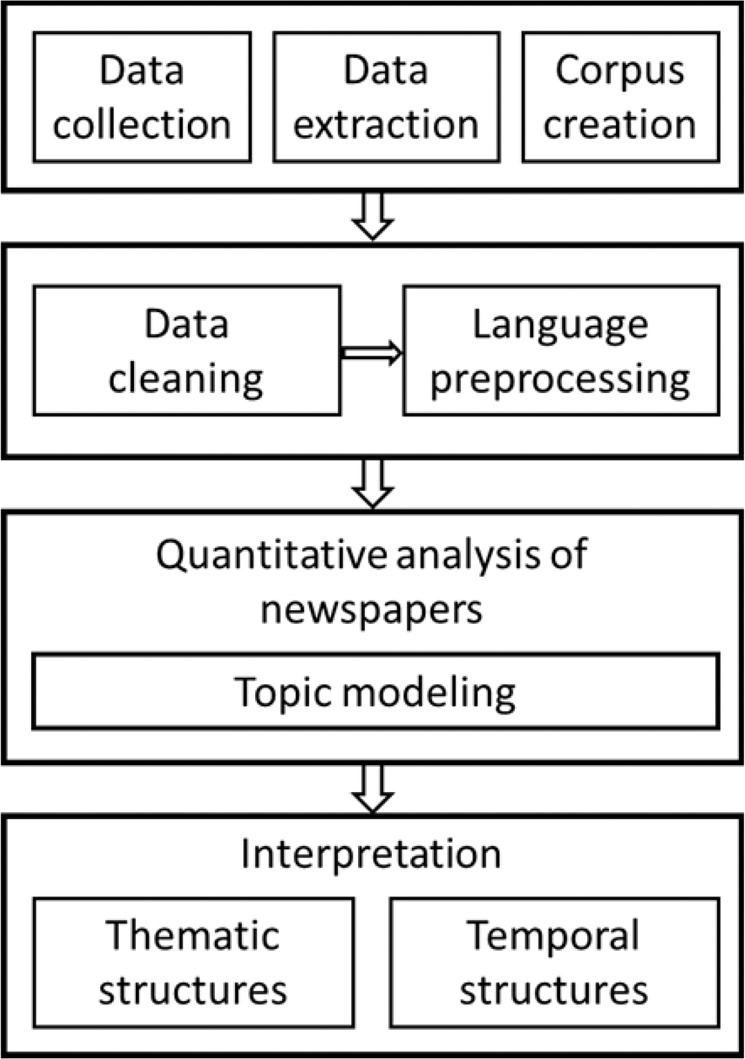
Methodological phases in the text mining process. Source: authors’ diagram; adapted from Gründer-Fahrer et al. (2018)

Figure 1 gives an overview of the analytical steps of the text mining process.

We searched for all articles in the digital archives of the four newspapers that contained the search term gülle, *gülle, gülle* and *gülle*. Slurry (Ger. Gülle) consists of the liquid and solid components of animal excreta, mixed with a low share of chopped straw. We extracted the articles and created a text corpus using the software package quanteda (Benoit et al. 2018) in the statistical program R (R Core Team 2019). We excluded articles in which the search term appears as a person or place name (güller, güllen).

A number of standard preprocessing steps served to standardize the words in the text corpus. The text was split into single words, punctuations, numbers, Unicode symbols and URLs were removed, and words were transformed to lower cases using the tm package (Feinerer et al. 2008). Inflected word forms were lemmatized to their lexical base forms, e.g., plural to singular or verbs to their nominal forms, and stop words were removed.

We identified bigrams—pairs of words that appear together significantly more often than statistically to be expected – and transformed the first 400 into single word units (cf. Benoit et al. 2018; Lau et al. 2013). We removed all tokens that appeared in less than 1% of all documents, and removed the ten most frequent words, which, due to their high frequency, made no meaningful contribution to revealing patterns. Furthermore, we created a separate custom list in order to unify subject-specific terms (e.g., “Slurry Ordinance” to “Fertilizer Ordinance”) and to convert abbreviations into the corresponding full terms (e.g., WFD to Water Framework Directive).

For the analysis of the text corpus, we used a Latent Dirichlet Allocation (LDA) model. LDA is a Bayesian probabilistic topic model that, by calculating statistical correlations of words, uncover semantic structures, so-called topics (Blei 2012). The model is based on the assumption that similar words occur in similar contexts based on the probability of co-occurrence. To our understanding, LDA topics reflect issues (Mempel and Corbera 2021) that are semantically framed in certain ways (Rychlý 2014). While topics are pure collections of terms, in issues, the terms gain meanings.

We used Gibbs sampling, a method based on a Markov chain Monte Carlo Algorithm that iteratively approximates the most likely co-occurrence of terms. A Gibbs sampler requires the specification of the desired number of topics as well as alpha and beta, the “parameters of the prior distributions” for topics over documents and words over topics (Grün and Hornik 2011). We chose 0.2 as alpha and 0.1 for beta as well as 20 topics after running the model with different values. In total, we ran 1000 iterations.

**Fig. 2:**
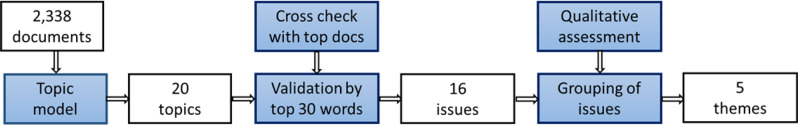
Methodological stages of the issue identification process

Figure 2 shows the process of how the issues were identified. For each of the 20 topics, we let the model compute the 30 words that most represent the topics. It is common in topic modeling that not all topics contribute to explain a phenomenon, in our case the representation of slurry (cf. Jelodar et al. 2019). Therefore, we qualitatively evaluated the 30 top words per topic and the top 10 articles in which the respective topic was most represented. Four topics were excluded from further analysis that were either too general (“federated state election campaigns: e.g., “green, party, cdu, spd, political, lower_saxony”) or not informative for understanding slurry representation of the media (literature: “book, novel, woman, read, live, write”; “drive, woman, people, think, ask”; crime and accidents: “father, police, woman, old, court, child”) (cf. DiMaggio et al. 2013). The four excluded topics accounted for 24.7% of the total corpus. This procedure resulted in the identification of 16 issues and five groups of issues, i.e., themes.

In order to validate the results, we analyzed the development of the issues over the investigation period in order to explore when the issues appeared on and disappeared from the media agenda or if they were covered continuously. We compared changes in coverage of slurry with potentially relevant events.

## Results

**Fig. 3:**
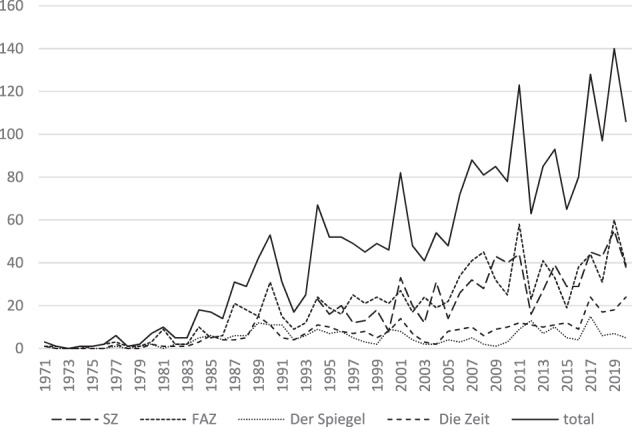
Development of media coverage related to the search term “slurry” from 1971 to 2020

Increasing political and public attention has been reflected in a rising coverage of slurry in the newspapers. Figure 3 shows how reporting developed during the investigation period. Attention related to slurry emerged in the late 1970s and coverage increased steadily over time. The text corpus of SZ only started in 1994 which caused a leap in the number of articles but did not distort the fact that journalistic attention increased overall. While the number of articles was almost zero in the 1970s, it rose to an average of 60 in the 1990s and peaked in 2019 with 140 articles.

### Issues Covered in the German Newspapers

Table 2 shows the 16 issues generated by the topic model. After discussion and joint agreement of the authors on their plausibility, we assigned short, qualitative labels to each topic that best represent their contents. Each issue is shown together with the 30 most frequently observed words, which were translated from German to English for better comprehension, as well as their percentage shares in the total text corpus.

**Table 2:** Issues generated by the Latent Dirichlet Allocation (LDA) model, Top 30 words, and shares

Label	Top 30 words	Share
Bioenergy	biogas plant, plant, biogas, electricity, biomass, energy, heat, gas, renewable energy, wind, generate, may, power plant, natural gas, use, kilowatt hour, megawatt, cent, renewable raw material, energy plant, bioenergy, produce, biodiesel, promotion, operation, cogeneration plant, energy transition, plant operator, power generation, grid	6.6%
Agricultural policy	eu, brussels, federal government, farmers association, european, berlin, money, rukwied, commission, politics, claim, klöckner, german farmers association, proposal, government, prospective, billion euro, europe, eu-commission, künast, schmidt, consumer, threaten, brussel, ministry, demand, law, agricultural policy, dispute	6.0%
Farm structural change	price, ecological, operate, production, agricultural policy, area, consumer, cost, market, product, agricultural, economic, preserve, farm, peasant, economy, subsidy, economic, system, state, necessary, small, environmental protection, environment, chance, animal husbandry, income, possibility, development, expensive	6.0%
Groundwater pollution	groundwater, nitrate, drinking water, threshold, nitrogen, soil, slurry, fertilizer, problem, fertilize, milligram, water, waterworks, nitrate pollution, pollution, liter, region, fertilizer ordinance, exceed, federal government, nitrate value, farmland, well, lower saxony, fertilization, pollute, spread, field, value, comply	5.9%
Local governance	community, city, hesse, project, mayor, county, hessian, citizen, build, municipality, district, plant, citizen initiative, company, resident, müller, public, frankfurt, darmstadt, city, utility, expert opinion, munich, plan, site, district administrator, build, participate, approval, project, urban	5.5%
Soil & air pollution	soil, contain, researcher, plant, nitrogen, air, scientist, phosphorus, process, methane, phosphate, kilogram, fertilizer, atmosphere, nutrient, emerge, ton, substance, utilize, bacterium, quantity, use, ammonia, carbon dioxide, sewage sludge, gas, organic, compost, greenhouse gas, chemical	5.2%
Bucolic countryside	village, house, city, old, live, small, place, kilometer, street, area, guest, forest, landscape, drive, region, church, border, wind, meter, tell, beautiful, dwell, move, resident, visitor, hotel, couple, cook, middle, field	5.1%
Business & innovation	company, corporation, euro, price, million euro, customer, market, business, sell, money, employee, branch, llc, bank, managing director, product, group, past, sale, buy, win, get, cost, entrepreneur, investor, build, expensive, manufacturer, employ, invest	5.1%
Globalization	world, global, worldwide, usa, europe climate change, problem, climate, study, political, china, important, american, international, million, environment, america, politics, car, climate protection, sustainable, humanity, ecological, state, industry, billion, earth, question, future, consequence	4.4%
Animal welfare	animal, pig, meat, stable, chicken, eat, beef, piglet, vegan, mass animal husbandry, consumer, sow, slaughterhouse, live, pork, slaughter, animal husbandry, produce, sausage, nutrition, hold, fattening, lower saxony, kilogram, eat, soy, supermarket, ban, million, animal rights activist	4.2%
Human health	antibiotic, germ, pathogen, bacterium, infection, patient, ehec, vegetable, case, infect, medicine, doctor, disease, cucumber, virus, dangerous, sicken, examine, animal, die, sample, hospital, resistant, researcher, resistance, week, frequent, sick, clinic, examination	3.9%
Farm management	soil, hectare, field, farmland, area, may, plant, harvest, conventional, grow, tractor, cultivation, grain, machine, yield, cultivate, wheat, organic, potato, ecologic, combine harvester, fertilizer, vegetable, plant protection product, farm, pesticide, organic farmer, harvest, use, weed	3.9%
Liberalization of markets	cow, company, milk, barn, dairy farmer, animal, calf, cent, dairy, sheep, milk cow, milking, price, liter, stove, liter milk, milk-price, farm, family, pasture, father, cheese, son, cattle, tractor, shepherd, get, farmer, bavarian, grass	3.8%
Biodiversity loss	nature, forest, tree, nature conservation, animal, bird, insect, conservationist, landscape, meadow, disappear, rare, plant, habitat, ecological, forester, soil, hunter, plant species, wild, national park, area, live, strong, harm, nature reserve, decade, beetle, hare, biologist	3.6%
Waterbody pollution	water, river, lake, sea, wastewater, waterbody, brook, sewage plant, fish, alga, north sea, drinking water, flow, toxic, fishing, chemical, bath, denmark, meter, million, baltic sea, bodensee, pump, broth, environmental protection, shore, liter, kilometer, swim, entitled	3.5%
Industrial agriculture	gdr, mark, federal republic, lpg, hectare, west german, million mark, east, west, reunification, village, east german, cooperative, old, socialist, saxony, netherlands, dutch, holland, van, mecklenburg-western pomerania, government, billion mark, soviet, bonn, former, sed, ton, pastor, animal production	2.7%

### Interpretation of Issues and Representations of Slurry

In order to answer RQ 1 and RQ 2, we interpreted the issues and representations of slurry derived from the topic model. In Table 3, the issues are compiled along five major group themes together with their temporal distributions and possible initiating events, actors cited in the top 30 terms, story lines, and representations of slurry. We chose labels that best described the issues of the respective themes, regardless of whether the terms were explicitly mentioned in the newspapers.

**Table 3:** Summary and interpretation of results: main period of coverage, triggering events, actors, story lines and representation of slurry

Label	Main period	Events/causes	Actors appearing in top 30 terms	Story line	Representation of slurry
*Structural change theme 16.8%*					
Farm structural change	Late 1970s – late 1980s	Permanently on the agenda	Consumer, state	Family farms are environmentally sound and support animal welfare	“Too much”, linked to factory farming
Liberalization of markets	2008/09, 2015	2008/09 low milk prices, 2015 removal of milk quota	Family, father, son, shepherd, farmer	Profitability of small and medium-scale farms decreases due to market liberalization	Milk is dumped in slurry pits – milk and slurry are metaphorically treated equally as waste
Animal welfare	Steady increase, yet with strong upward and downward fluctuations from year to year	1996 professional ban of a large-scale chicken breeder because of cruelty to animals and violation of food law; 2001 pig scandal in Bavaria due to feeding of drugs for faster fattening; peak of BSE crisis; 2020 African swine flu	Consumer, animal rights activist	Disastrous housing conditions for animals – in recent years processors, retailers and consumers to blame	Poisonous waste that contaminates ground and drinking water and over-fertilizes farmland
Industrial agriculture	Mid 1970s to 1993, insignificant thereafter	1989/90: Germany’s reunification; afterwards (often Dutch) investors’ factory farm construction activities	Government, SED*, pastor, Dutch (*Socialist Unity Party of Germany; state party of GDR)	Industrial (animal) production and its impacts on the environment, particularly water, as well as social structures	Excessive accumulation of slurry contaminates soil and drinking water
*(Un)sustainability theme 22.1%*					
Surface water pollution	Slight decrease over the investigation period	–	–	Impacts of human behavior on water bodies and biodiversity	Excessive accumulation and discharge into waterbodies
Groundwater pollution	1982–1985 and 2014–2020	2014–2020 implementation of Fertilizer Ordinance	Federal government	High nitrate values lead to water pollution and cleaning costs, EU law demands compliance	Too much slurry on the fields pollutes groundwater
Soil & air pollution	Slight decrease	2001 UN Climate conference (Bonn)	Researcher, scientist	Fact of pollution and (scientific) solutions for combating pollution of natural resources (particularly air and soil) from slurry	Slurry is transmitter of harmful substances
Biodiversity loss	Minor decline over investigation period, peaks in 1983, 1987 and 1996, slight peak in 2001	No significant events; 2001 forest health report	Biologist	Intensive agriculture harms fauna and flora: nitrogen surplus, pesticides and monocultures lead to species displacement, nitrogen in air supports forest dieback	Too much slurry causes nitrogen surplus and endangers biodiversity
Human health	Peak in 2011	EHEC, sporadically MRSA, other diseases negligible	Researcher	Farming practices are suspected to cause health problems	EHEC bacteria: contaminated slurry threatens consumers; antibiotic resistant MRSA bacteria threatens human health: factory farming is responsible due to excessive application of antibiotics
*Governance theme 15.9%*					
Globalization	Steady increase	2001 Climate conference (Bonn); 2005 ratification of Kyoto protocol, 2015, 2017, 2019 Climate conferences	USA, Europe, America, humanity, industry	Globalization leads to industrial agriculture and emissions, which hamper equitable distribution and worsens resilience	Reference to nitrate, endangers environment, particularly groundwater and climate
Agricultural policy	Steady increase, disproportionally high in the 2010s	–	EU, federal government, farmers association, Rukwied*, commission, Klöckner**, German farmers association, EU-commission, Künast**, Schmidt**, consumer, ministry (*president of German farmers association; **former agricultural ministers)	Failure of agricultural policies to protect nature and environment, conflicts between stakeholders	Too much slurry on the fields
Local governance	Steady decrease	–	Mayor, citizen, resident, Müller, public, district administrator	Citizen involvement, resistance to slurry-related buildings	Waste that degrades quality of life of residents, often related to factory farms
*Bioeconomy theme 15.6%*					
Farm management	Stable with minor peaks	–	Organic farmer	Sustainable agriculture can be achieved by use of technology	A substance, which is managed by technology
Bioenergy	2002–2012	Energy transition, legislation for bioenergy (EEG)	Plant operator	Bioenergy is a sustainable alternative to fossil energy to master the German energy transition	Substrate (input) for bioenergy production
Business & innovation	Approx. 2006–2013 – time period analogous to *bioenergy issue*	–	Company, corporation, customer, employee, managing director, entrepreneur, investor, manufacturer	Technology can contribute to transform slurry in useful monetizable products	A substance that can be exploited by technology to generate profit
*Countryside theme 5.1%*					
Bucolic countryside	Stable over entire period	–	Resident, visitor	The “scent” of slurry is a characteristic of the bucolic countryside	Smell is unpleasant but inseparable from the countryside

All but four issues used a negative representation of slurry (e.g., “poisonous waste”). In the bucolic countryside issue slurry was framed in a negative way (Table 3) but embedded in a positive story line of pleasant country life (“scent of countryside”). Only the three issues of the bioeconomy theme were represented in a neutral or positive way (e.g., “raw material for biogas production”).

### Structural Change Theme

The structural change theme comprises four issues: (i) farm structural change, (ii) liberalization, (iii) animal welfare, and (iv) industrial agriculture. It deals with the transformation of the German farm structure and related social problems. For example, the demise of small and medium-size farms as well as low producer prices for agricultural products were frequently discussed. The newspapers predominantly conveyed a positive image of family farms associated with ecological soundness.

Slurry was mostly constructed as dangerous waste that emerged when livestock was kept in industrial farms, while family farms were not associated with typical slurry-related externalities. The newspapers related factory farming to (the demand for) cheap meat and human health. Over the investigation period large-scale slaughterhouses, retail chains and consumers were increasingly held responsible for the price pressure on farmers. An example is extensive reporting on the collapse of the milk price in 2008, when farmers poured the produced milk in their slurry pits, equating society’s appreciation of milk to that of slurry. Rising criticism from various non-governmental groups about global production and supply chains accelerated coverage in recent years.

Socialist agriculture in the former German Democratic Republic (GDR) in East Germany served as a negative example: newspapers argued that the GDR’s strategy of separating arable and livestock farming and producing in ‘agricultural production cooperatives’ (Ger. Landwirtschaftliche Produktionsgenossenschaften, LPG) resulted in industrial agriculture, factory farming in large production facilities, alienation and social erosion. After the reunification of the GDR and the Federal Republic of Germany (FRG) in 1989/90 foreign investors purchased large areas of farmland and constructed factory farms which was critically addressed for land grabbing and furthering structural change and industrial agriculture.

### (Un)sustainability Theme

The (un)sustainability theme comprises five issues that cover emissions from slurry and its impacts on the environment, water, biodiversity, and humans: (i) surface water pollution, (ii) groundwater pollution, (iii) soil & air pollution, (iv) biodiversity loss, and (v) human health. Here, slurry was represented as a substance whose excessive accumulation leads to discharge to water and soils and subsequently endangers biodiversity and human health.

Only in this theme, the newspapers referred to scientific expertise to a significant extent. Particularly in the soil & air pollution issue research efforts and technologies to overcome emissions into the atmosphere and discharges into the soil from slurry application were discussed.

The other three issues were framed in a problem-oriented way: excessive application of slurry and mineral fertilizers results in the pollution of surface and drinking water and poses a threat to human health. Accumulation of slurry by factory farms, pesticide application and agricultural monocultures were held responsible for decreasing biodiversity. Slurry as a means of transportation of dangerous diseases and medicals found journalistic attention, e.g., the excessive treatment of animals in intensive livestock farms with antibiotics. In May 2011 the clustered spreading of EHEC, a pathogen strain of the intestinal bacterium Escherichia coli, was extensively covered by the newspapers when people died after consumption of vegetables and the bacterium was – unjustifiably – suspected to be transmitted via organic fertilizers.

### Governance Theme

Within the governance theme, three policy-related issues which focus on different administrative levels were on the agenda of the newspapers: (i) local governance, (ii) agricultural policy and (iii) globalization. Slurry was mainly discussed in terms of excessive accumulation on farmland due to policy failure that leads to threats to the environment and the climate. At the local level the newspapers illuminated concrete conflicts between farmers and citizens and, fewer, positive coverage, e.g., about bioenergy for sustainable energy supply. At the national and European Union level negotiations between politics and the agricultural lobby prompted discussion about the effects of policy instruments and reforms. The globalization issue was discussed in terms of a global problem whereby globalization was partly held responsible for the industrialization of agriculture. Policy at the global level was related to scientific expertise and distributional justice in the global society.

### Bioeconomy Theme

The bioeconomy theme has been referred to since the 1990s and was addressed in a solution-oriented and techno-economic way. Slurry was seen as biomass that could either be managed with technology or produced, exploited and used. Accordingly, the bioeconomy theme comprises three issues, in which slurry is represented as a resource: (i) farm management, (ii) bioenergy, and (iii) business & innovation.

The farm management issue was oriented towards innovations for sustainable agriculture. Journalistic attention shifted from organic farming towards smart farming and digitization in agriculture since the mid-2010s. Bioenergy was constructed as a sustainable, environmentally friendly alternative to fossil energies. Media retold the German government’s discourse of an “energy transition” with renewable energies that would contribute to climate protection. Slurry was represented as a raw material for energy production. The newspapers covered technical innovations that could contribute to transform slurry into marketable products in an environment of economy and entrepreneurship.

### Countryside Theme

Finally, the bucolic countryside issue builds a theme on its own. The newspapers predominantly communicated a positive picture of rural life in which slurry was an indispensable, though annoying element of country life. Feuilleton articles with landscape and travel descriptions prevailed. Slurry was one element for telling the actual content of a story, i.e., used in a wider context, e.g., about visits to the countryside where “the scent of slurry” was in the air.

### Comparison of Newspapers

The four newspapers had different numbers of articles (cf. Table 1). We, therefore, calculated the percentages of each newspaper on every single issue. We found for all issues coverage of at least 1.6%, though with different foci (cf. Table 4). Overall, deviations were not significant.

**Table 4:** Foci of the newspapers with regard to the issues

	Structural change				(Un)sustainability					Governance			Bioeconomy			C*
	family farm	Liberalization	Animal welfare	Industrial agriculture	Waterbody pollution	Groundwater pollution	Soil & air pollution	Biodiversity loss	Human health	Local governance	Agricultural policy	Globalization	farm management	bioenergy	business & innovation	bucolic countryside
FAZ	7.1	3.5	3.3	2.6	2.8	5.4	6.1	3.5	3.4	7.4	5.0	3.5	4.6	7.7	5.4	5.2
SZ	5.0	4.8	4.2	1.6	3.9	7.0	4.1	3.7	4.6	4.3	8.5	4.6	3.3	7.4	5.0	4.3
Spiegel	4.4	2.9	5.5	5.5	5.1	5.6	5.1	4.1	4.7	4.5	5.2	4.3	2.8	4.0	4.7	4.9
Zeit	5.8	3.4	6.0	3.5	3.4	3.7	5.1	3.5	3.4	3.2	4.3	6.6	3.6	3.7	4.6	6.7

C* Countryside theme

The two daily newspapers SZ and FAZ were more active in episodic reporting on real-world events (cf. Behr and Iyengar 1985). They preferentially covered the bioenergy issue directing to practical topics. FAZ was also strongly devoted to the local governance issue. SZ was additionally active in the agricultural policy issue and the groundwater pollution issue. It also covered the liberalization issue (removal of milk quota); this focus partly reflects the farm structures in Southern Germany (the original region of SZ) with an above average number of small and medium-size farms and their concerns.

While the daily newspapers were able to react timely on recent events weekly newspapers had higher shares of thematic articles. They tended to cover issues at the national and international level. Zeit particularly engaged in the animal welfare issue and the globalization issue. On the other hand, Zeit published feuilleton articles related to the bucolic countryside issue, e.g., travel reports. Spiegel focused on issues that covered degradation of nature and environmental resources. It was the one that covered most strongly the industrial agriculture issue and was the greatest critic of GDR agriculture and its impacts on the environment.

### Temporal Distribution of Issues

**Fig. 4:**
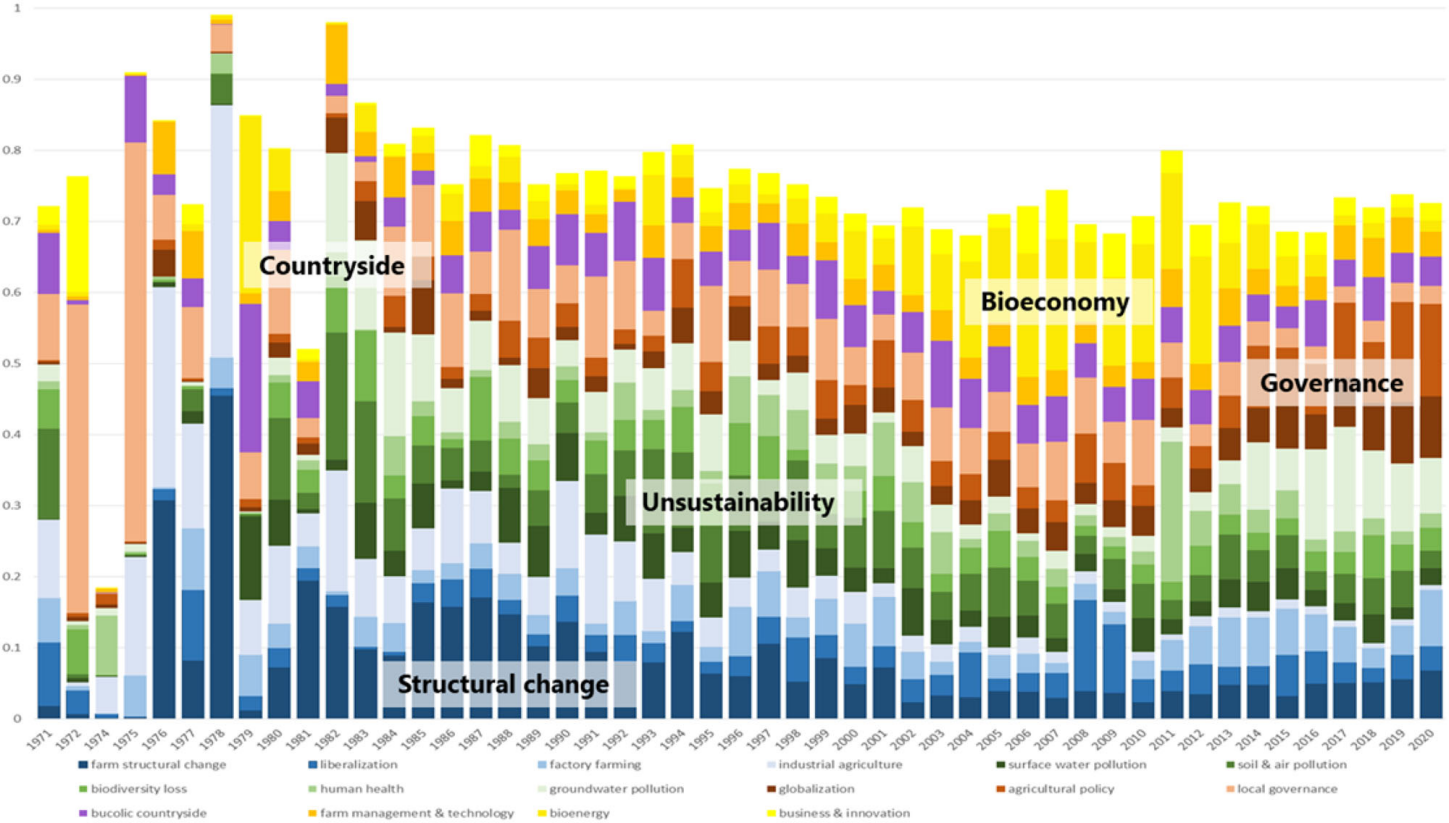
Development of shares of issues from 1971 to 2020 (blue = structural change theme, green = (un)sustainability theme, brown = governance theme, violet = bucolic countryside, yellow = bioeconomy theme)

In order to answer RQ 3 and RQ 4, we traced the development of issues and themes over the investigation period. The development of the five themes is illustrated by means of a color scheme in Fig. 4. High deflections in the 1970s are due to small numbers of articles in the individual years. The structural change theme was of high relevance in the 1970s and 1980s and decreased in the 1990s; yet, with a slight upward trend in recent years. The (un)sustainability theme decreased after a longstanding high in the 1990s and 2000s with a rash in 2011 due to EHEC and an increasing share since 2016 due to a greater attention to the groundwater pollution issue. The bioeconomy theme strongly declined after a longstanding high in the 2010s, for which the strongly decreasing coverage of bioenergy was responsible. The governance theme remained largely constant, yet, with a shift from local governance to German and EU agricultural policy and globalization. Also, the countryside theme stayed largely stable.

For external validation of the topic model results, we compared the newspapers’ reporting with events that were (potentially) relevant to the salience of issues (cf. Quinn et al. 2010). Two issues show clear peaks that were due to concrete single events indicating episodic coverage. First, the human health issue spiked in 2011 due to the EHEC outbreak. Second, the liberalization issue peaked in 2008/2009. Record-low prices in 2008 prompted the farmers to pour away the produced milk in their slurry pits in order to denounce the disrespect of society.

Two issues were covered intensively over a longer time period followed by a decrease. First, the industrial agriculture issue focusing on the GDR socialist agricultural system and its impacts on the environment was discussed until 1990. After Germany’s reunification, this issue largely left the agenda. Second, the bioenergy issue was increasingly covered from 2000 to 2012 with the implementation of the national “Renewable Energy Act” (Erneuerbare-Energien-Gesetz) in 2000 and the EU “Renewable Energy Directive” 2009/28/EC in 2009. With the amendment of the “Renewable Energy Act” in 2014 the compensation for electricity fed into the grid was reduced and the construction of new plants decreased considerably, and, with this, the coverage of the bioenergy issue decreased. After several one-off peaks, the business & innovation issue had a longer high phase from 2006 to 2013, similar to the bioenergy issue.

The share of the local governance issue considerably decreased since 2010. The share of articles of the surface water issue also showed a steady decrease in the investigation period from 12% in 1979 to 2.5% in 2020. In both cases, we could not identify an unambiguous occasion that was responsible for the decline in interest.

The animal welfare issue was relatively evenly distributed over the investigation period with renewed intensity in coverage in 2020. In 2019 the then CDU-led Federal Ministry of Food and Agriculture initiated the notification of a voluntary animal welfare label that heated again the public debate and media coverage of factory farming and excessive accumulation of slurry.

In the 2010s the agricultural policy issue together with the groundwater pollution issue gained in journalistic interest. The EU lawsuit for failure to implement the Nitrates Directive by the German federal government and several amendments of the Fertilizer Ordinance prompted the newspapers to thematic coverage. Around 2019, the realignment of the EU’s Common Agricultural Policy (CAP) also triggered coverage of its relevance for, among others, slurry accumulation.

The share of the globalization issue steadily increased in the investigation period and partly displaced the soil & air pollution issue. The upwards trend of the globalization issue and its peaks partly correspond to the outcomes and controversies of the climate conferences, such as the 2001 climate conference in Bonn, Germany, the 2005 ratification of the Kyoto protocol, its follow-up protocol in Paris in 2015, the Bonn climate conference in 2017 as well as the 2019 conference in Madrid whose results were sharply criticized by non-governmental organizations and discussed in the media.

## Discussion

### Issues, Meta-Themes, and Representations of Slurry

Our analysis shows a wide range of thematic emphases related to livestock slurry that were set by the four leading German newspapers. Articles were not solely focused on agriculture as could be assumed from the fact that slurry is a by-product of agriculture. Instead, they reached from purely agricultural topics (e.g., technical innovations in the farm management issue) to global societal problems, such as climate change, and from directly addressing slurry (e.g., bioenergy issue with slurry being an input for producing renewable energy) to a critical discussion of social and political grievances (e.g., farm structural change).

Despite the multitude of issues and representations of slurry, we found in most issues a latent underlying pattern that reflects the Germans’ leitmotif of agriculture. Media coverage of slurry turned out to reflect public discourse of a socially just, environmentally sound and economically viable agriculture that stewards the rural landscape, the rejection of industrial agriculture with its negative impacts on the landscape, nature, environment, climate, animal welfare and health, and the responsibility and failure of policy for the enforcement of social preferences. Exceptions are the issues of the bioeconomy theme that focused on the (sustainable) use of slurry as a valuable resource.

While our original intention was to investigate coverage of slurry as a topic per se we found that only six issues were directly dedicated to slurry in a considerable number of articles. Slurry was the core topic in many articles of the bioenergy issue and the business & innovation issue as being an input for bio-economic innovations and in the human health issue when slurry was assumed to be carrier of EHEC bacteria. The soil & air pollution issue directly referred to slurry when slurry pollution and technologies for reduction were covered, and in the groundwater pollution issue slurry was mainly discussed in the context of the amendments of the German Fertilizer Ordinance. In the miscellaneous local governance issue one topic which directly referred to slurry was the resistance of individuals against the construction of stables or other agricultural facilities, because of odor nuisance or noise, known as “Not In My Backyard” (NIMBY) syndrome (Furuseth and O’Callaghan 1991). All other issues contained slurry as part of a story line to produce salience in a preferred way.

We supposed a neutral representation of slurry in the beginning of the investigation period when slurry-based housing systems were less widely adopted (Settele 2020). In contrast, we found that since the beginning of coverage the tone was mostly critical. The initially low number of articles could mean that the topic was of little interest at that time and only emerging. Media coverage related to slurry intensified in the 1980s likely due to further intensification of livestock systems and the accrual of more slurry. Coverage steadily increased in the following decades suggesting a rising societal problem awareness of the negative impacts of livestock farming and as such, a sign for societal dissatisfaction with agricultural practices and political environmental action. In most issues slurry was represented in a negative way, for example as dangerous waste that over-fertilizes fields, pollutes water (eventually contaminated with bacteria, antibiotics, etc.), degrades the environment and biodiversity, and contributes to climate change.

Yet, in four issues (bioenergy, business & innovation, farm management, soil & air pollution) it was suggested that problems related to excessive accumulation of slurry could be addressed with technical solutions. Since the 2010s a new tentative attribution of slurry as a resource for the bioeconomy emerged, triggered by Germany’s energy transition discourse (cf. Kaiser et al. 2016) but lost again in significance since the 2014 amendment of the Renewable Energy Act.

These results led to the identification of three meta-themes that were latently present in a large part of slurry-related reporting over the entire investigation period: (i) the dichotomy of agricultural industrialization and family farming; (ii) contrasting actualities of factory farming and animal welfare; and (iii) the responsibility of policy for the emergence, existence, and solution of livestock and slurry-related problems.

These meta-themes reflect the dichotomy between small-scale, family-operated farms and industrially driven agriculture: while society holds on the image of peasant farming, industrial farms expand and displace or absorb them. It was mainly highlighted that family farms operate in an environmentally and animal friendly way, and are threatened by agricultural policy whose design favors large farms and paves the way for industrial agriculture and intensive animal husbandry.

Our results show that media coverage of the three meta-themes largely corresponded with the public agenda. Theuvsen et al. (2017), for example, in a survey with 1150 German citizens found that 80% preferred peasant farms as they manage better in harmony with the environment. Interviews with participants of the annual demonstration organized by the German movement “Wir haben es satt!” (“We are fed up!”), which promotes a sustainable, fair agriculture and food production were even in line with most issues covered by the newspapers (Meinecke et al. 2021). Reporting equally corresponds to the political-scientific discourse in Germany. In 1984, for example, the German Environment Agency published a report on the structural change in agriculture, in which the “modern, intensified and highly specialized agriculture” is criticized for its environmental damages (Umweltbundesamt 1984), and, 30 years later, the Scientific Advisory Board on Agricultural Policy (2015) pointed out the need to create “pathways to a socially accepted livestock husbandry in Germany”.

Political scientists argue that the advent of public environmental awareness in the 1960s and 1970s and the formation of new actor groups led to the transition of politics from ‘exceptionalism’ based on “compartmentalized, exclusive and producer-centered policies and politics” to ‘post-exceptionalism’ with non-exclusive, complex and contested policies (Daugbjerg and Feindt 2017). Yet, we found in the analyzed newspapers that the media focused on traditional political institutions of national and EU government and the German farmers’ association when they addressed the responsibility of policy for the emergence, existence and solution of livestock and slurry-related problems. The newspapers highlighted cuts in subsidies which forced farmers to increase turnover and performance and either become industrially operating farmers or abandon the farm. The phrase “grow or die” coined by Sicco Mansholt, agricultural commissioner of the European Economic Community in 1968 is still used in the newspapers^1^. Another example is the removal of the EU milk quota in 2015 that prompted the newspapers to discuss it in the light of competition and the imperative to more intensive farming: “the modern cow” is “bred for high performance, finely maintained and cleverly tuned”^2^. Here, a single event was set in a larger context of societal concerns about industrialization of agriculture (Feindt 2010; Spiller and Theuvsen 2009). Yet, in the last two decades responsibilities for industrial agriculture and intensive animal farming were increasingly ascribed to consumers, processors and retailers by the press. This was discussed in the context of all three meta-themes and appear in the top words of the farm structural change issue, the animal welfare issue and the agricultural policy issue.

In research and farming practice techno-centric digital solutions have taken hold in the last decade, such as more efficient and resource-saving technologies (e.g., precision fertilization using near-infrared spectroscopy or satellite images; cf. Artner-Nehls and Méité 2022; Prause et al. 2020). Schmidt (2018), for example, argues that an approximation of large conventional farms towards sustainable agriculture and increased production can be expected. The discursive turn of science and practice towards larger, resource-efficient farms based on digital solutions was increasingly reflected in the farm management issue since 2013. While the FAZ articles rather promoted the use of technological solutions for efficient farming the other newspapers highlighted the critical points of digital farming, such as data security and market concentration of suppliers. Spiegel, moreover, warned of structural change of agriculture through digitization^3^ and, thus, unmasked the techno-optimistic framing of food production as contradictory to society’s preferences.

### Events and Issue Prevalence

In accordance with Iyengar (1991), we identified both, episodic and thematic reporting. Within this categorization, we could differentiate between ‘short-term’ episodic (e.g., health issue) and ‘long-term’ event-oriented news (e.g., bioenergy issue) that consist of a string of ever new events that merged to one long-term issue on the media agenda. The identification of such long-term, though event-oriented, issues match with the fundamental research of Galtung and Ruge (1965) who found that an issue is more likely to stay on the media agenda once it appears in the news. Especially the daily newspapers reported on smaller events with a regional focus, in particular with regard to the local governance issue. Other issues, in turn, could rather be characterized as thematic issues that eventually may hook on smaller events.

With regard to episodic news, media coverage and events are generally well aligned. First, we found issues with peaks in a short period, such as the outbreak of EHEC in 2011 or the milk price crisis in 2008/2009. Second, we found issues that were intensively covered for some years but lost significance aftermath, such as the bioenergy issue whose guiding theme ‘energy transformation’ was fueled by ever new political events, such as the reduction of the compensation for electricity fed into the grid and the decline in new plant constructions (Matthes 2018) but lost its news value in the end. The bioenergy issue was in this period even so strong that it outweighed all other issues, and thus also the meta-themes. The industrial agriculture issue almost vanished from the media agenda after the German reunification in 1989/90 when reporting about GDR agriculture became obsolete. In the case of the groundwater pollution issue intensive coverage in the 1980s was followed by a downturn until 2013 and a steep rise thereafter which is due to a longstanding dispute about the implementation of the EU Nitrates Directive in Germany which resulted in proceedings of the European Commission against Germany because of non-compliance.

Third, we found relatively consistent thematic reporting throughout the investigation period, e.g., the waterbody pollution issue or the biodiversity loss issue, often related to small events, such as a scientific report or the construction of a large fattening farm. It can be assumed that these issues are based on perpetual problems which is why they were permanently present in the newspapers.

Fourth, there is a number of thematic issues whose significance increased or decreased throughout the investigation period: the agricultural policy issue and the globalization issue increased in significance while the farm structural change issue and the local governance issue decreased. Yet, we found a slight upwards trend of the farm structural change issue, together with the animal welfare issue, possibly connected to the recent recognition of the consumers’ co-responsibility for the status and development of the farming sector in Germany.

The thematic issues covered by the newspapers can be related to political, scientific, and public discourses, such as globalization or digitization in agriculture. These issues may result from uptake or critical reflection of existing discourses by journalists suggesting that the media and the audience interact in the construction of reality as proposed by McQuail (1994). In this context, we found a shift from the soil & air pollution issue to the globalization issue. The soil & air pollution issue had similar content as the globalization issue, yet, a different story line. In the 1990s the focus was on the impacts of factory farming on the climate while at present mass animal husbandry is identified as only one among diverse drivers. The place of the story line shifted from Germany and the EU to the global level, and the issues shifted from solution-orientation to problem-orientation. Most of the top articles of the soil & air pollution issue were about scientific research or technologies to overcome the problems of emissions from slurry. The story line of the globalization issue was more complex, involving questions of the responsibility of industrial countries, of the resilience of agriculture and global distribution of food.

### Methodological Reflections

Topic modeling proved useful for analyzing media coverage of slurry and identifying issues that were set in relation to slurry in a comparatively large and unstructured text corpus. News sources increasingly make their text corpora available for quantitative analyses. Thus, our method is timely and meets these changing framework conditions. It also overcomes certain limitations of qualitative social science approaches.

Hence, topic models not only provide quantitative information, such as the probability of co-occurrence of terms but also qualitative information, as the joint appearance of terms reveals meaningful content. Additional qualitative studies can be used to study selected results in-depth and to provide information on framing and discursive strategies of newspapers. Furthermore, text mining of news sources may be a lower cost alternative to surveys on public opinions (e.g., Theuvsen et al. 2017), assuming that media coverage corresponds to the public attitudes as McCombs and Shaw (1972) suggest in their seminal study. Thus, qualitative and quantitative approaches as well as manual and computer-assisted methods can be seen as complementary approaches to studying societal discourses, which can be used for mutual verification.

### Implications

Identifying issues and underlying patterns related to slurry may show the media’s influence on and reflection of the public agenda with regard to slurry and how it changes over time. Our newspaper analysis contributes to an improved understanding of how the status quo evolved and reveals possible entry points to initiate changes. The story lines constructed around “slurry” by the media, and their interweaving with a variety of agricultural and environmental challenges, reveal a multi-faceted societal debate.

At the same time, an underlying homogenous societal perspective, reflected in the identified meta-themes, suggests core obstacles to change. Similarly, the long-term presence of several issues in the media is an indication for resistance to change, both by society and legal political institutions. Indeed, despite long-term criticism slurry-based farming systems are prevailing, and still promoted and expanded on other continents (Zhang et al. 2021).

Our analysis reveals a strong divide between agricultural producers and the society. Future challenges for farmers such as structural change, mandatory emission reduction, animal welfare, or biosafety are set against their livelihoods and social status that are strongly coupled to farming. On the other hand, society is burdened by negative externalities from livestock production, while, in their role as consumers, the majority demands sufficient, safe and affordable livestock products, but is often not willing to pay higher prices (cf. Statista 2022). A media coverage that constructs and reinforces a “thinking in camps” (“they” against “us”) is a potential obstacle to change. Instead, a more balanced recognition of perspectives and arguments by the media could reconcile societal, consumer and producer interests and thus help overcome mutual alienation and stagnation.

## Conclusions

From our analysis, we draw the following conclusions.

First, our analysis reveals a wide array of issues related to slurry taken up by the newspapers. They range from technological innovations over environmental issues to social problems, and from the local to the global level. Furthermore, they reflect either a solution- or a problem-oriented viewpoint. We identified 16 issues by use of an LDA topic model and grouped them in five themes: bioeconomy, farm structural change, (un)sustainability, governance, and bucolic countryside.

Second, the newspapers represented slurry in two ways: either it was the central topic of coverage, or it was only one element of a story, essential for supporting salience of certain aspects. The representation of slurry ranged from mostly critical (‘poisonous substance’) to few positive perspectives (‘resource for the bioenergy production’).

Third, episodic short-term reporting contrasted with long-term episodic and thematic reporting. Emerging issues were equally present as decreasing issues and issues constantly covered throughout the investigation period. Episodic reporting reflects key events in the last 50 years, particularly the German energy transition policy, taken up increasingly since 2002 but dropped in 2013 in anticipation of the 2014 amendment of the ‘Renewable Energy Act’. Impacts of unsustainable use or disposal of slurry on the environment were, in turn, constantly represented on the media agenda.

Fourth, we identified three higher-level meta-themes, i.e., dominant implicit frames that overarched the majority of issues, running through the reporting of slurry-related issues: (i) the dichotomy of agricultural industrialization and family farming; (ii) contrasting actualities of factory farming and animal welfare; and (iii) the responsibility of policy for the emergence, existence and solution of livestock and slurry-related problems. These meta-themes suggest that the newspapers reproduce the public discourse of the environmentally and animal friendly family farm situated in a bucolic landscape.

Overall, the broadness of media coverage, the long-term persistence of several issues and the reference to public discourse on German agriculture uncover contrasting public and producer interests. This gap reveals a “thinking in camps”, which is an obstacle to a sustainable transformation of the livestock system. But it also highlights a possible entry point: a more balanced recognition of both perspectives in the media could contribute to a reconciliation of consumer and producer interests, and thus support sustainable solutions that are acceptable to both farmers and society.

## Data Availability

Restrictions apply to the availability of these data. Data were obtained from *Frankfurter Allgemeine Zeitung* via purchase of data set. Data from *Süddeutsche Zeitung*, *Der Spiegel* and *Die Zeit* are available upon subscription.

## Footnotes

1 Süddeutsche Zeitung: Hegen und pflegen, 17 September 2017

2 Der Spiegel: Der Mensch macht’s, 16 February 2014

3 Der Spiegel: Apps für den Acker, 13 October 2017
